# Gene Expression Profiles from Formalin Fixed Paraffin Embedded Breast Cancer Tissue Are Largely Comparable to Fresh Frozen Matched Tissue

**DOI:** 10.1371/journal.pone.0017163

**Published:** 2011-02-11

**Authors:** Lorenza Mittempergher, Jorma J. de Ronde, Marja Nieuwland, Ron M. Kerkhoven, Iris Simon, Emiel J. Th. Rutgers, Lodewyk F. A. Wessels, Laura J. Van't Veer

**Affiliations:** 1 Divisions of Experimental Therapy, Molecular Biology, Surgical Oncology, The Netherlands Cancer Institute, Amsterdam, The Netherlands; 2 Central Microarray Facility, The Netherlands Cancer Institute, Amsterdam, The Netherlands; 3 Agendia BV, Amsterdam, The Netherlands; Ohio State University, United States of America

## Abstract

**Background and Methods:**

Formalin Fixed Paraffin Embedded (FFPE) samples represent a valuable resource for cancer research. However, the discovery and development of new cancer biomarkers often requires fresh frozen (FF) samples. Recently, the Whole Genome (WG) DASL (cDNA-mediated Annealing, Selection, extension and Ligation) assay was specifically developed to profile FFPE tissue. However, a thorough comparison of data generated from FFPE RNA and Fresh Frozen (FF) RNA using this platform is lacking. To this end we profiled, in duplicate, 20 FFPE tissues and 20 matched FF tissues and evaluated the concordance of the DASL results from FFPE and matched FF material.

**Methodology and Principal Findings:**

We show that after proper normalization, all FFPE and FF pairs exhibit a high level of similarity (Pearson correlation >0.7), significantly larger than the similarity between non-paired samples. Interestingly, the probes showing the highest correlation had a higher percentage G/C content and were enriched for cell cycle genes. Predictions of gene expression signatures developed on frozen material (Intrinsic subtype, Genomic Grade Index, 70 gene signature) showed a high level of concordance between FFPE and FF matched pairs. Interestingly, predictions based on a 60 gene DASL list (best match with the 70 gene signature) showed very high concordance with the MammaPrint® results.

**Conclusions and Significance:**

We demonstrate that data generated from FFPE material with the DASL assay, if properly processed, are comparable to data extracted from the FF counterpart. Specifically, gene expression profiles for a known set of prognostic genes for a specific disease are highly comparable between two conditions. This opens up the possibility of using both FFPE and FF material in gene expressions analyses, leading to a vast increase in the potential resources available for cancer research.

## Introduction

Tissue samples collected during surgery as well as biopsies are often fixed in Formalin and embedded in Paraffin (FFPE). Molecular genomics assays on archived FFPE blocks, together with clinicopathological information, can provide critical insights into a heterogeneous disease like breast cancer, especially considering the fact that FFPE samples are the most widely available source of tissue material for which long-term clinical follow-up data are recorded. The ability to perform gene expression profiling on these samples will enable many prospective and retrospective studies to be performed facilitating the association of expression profiles with clinical outcomes [1], [2].

Microarray expression analysis using FFPE tissues has been problematic as the retrieval of RNA from FFPE material is challenging [3]. FFPE archival methods lead to chemical modifications (methylene dimerization or monomethylolation) and the partial degradation of the RNA (up to 50% of the RNA may not contain an intact poly-A tail) [3], [4], making RNA extraction, reverse transcription and quantitation a difficult process [3]. In spite of these limitations, some studies have reported usable gene expression data from FFPE specimens with conventional microarrays technologies [3], [5]–[7]. Nevertheless, it is becoming apparent that protocols specifically designed for RNA extracted from FFPE tissues in microarray experiments can improve the quality of FFPE gene expression profiles [8]–[13]. For example, the addition of random primers to the cDNA synthesis reaction showed higher gene detection from FFPE than compared to oligo dT priming alone [14].

Recently, Illumina has developed an innovative assay called DASL (cDNA-mediated Annealing, Selection, Extension and Ligation) with the specific aim of overcoming the technical limitations that are associated with microarray-based analyses of FFPE samples. The DASL assay employs priming with random hexamers in the cDNA synthesis stage and therefore does not depend on poli-A/oligo-dT based priming. Moreover, the assay requires only short target sequences (about 50 nucleotides) for query oligonucleotide annealing implying that also degraded RNA can be quantified [2], [8]. The first DASL assay was limited to 1536 probes targeting 502 cancer related genes [8]–[10], but Illumina has extended the assay to include 24526 well-annotated transcripts. The Whole-Genome DASL Assay (WG-DASL Assay) allows for genome-wide profiling in archived materials [2] opening up new possibilities in cancer research.

To date there have been few publications on the application of the Illumina WG-DASL Assay. A study performed by Illumina evaluated the assay reproducibility and various technical aspects [2] and a more recent paper reported on an optimized protocol for sample preparation [15]. Another group has recently investigated the WG-DASL assay showing that it is possible to identify the molecular subtypes of FFPE familial breast tumors, but the study did not focus on the direct comparison of FFPE and Fresh Frozen (FF) pairs in terms of whole gene expression profiling [16].

Therefore, there is a need for a more systematic evaluation of the assay specifically focusing on the comparison of mRNA expression profiles obtained from FFPE material to those obtained from FF material, which has so far been the preferred source of mRNA for microarray profiling.

Currently, several prognostic microarray gene expression signatures for breast cancer have been generated using fresh frozen material [17]–[25] and one of them is being validated in a prospective trial [26]. Testing the reproducibility of these signatures on FFPE material represents a further assessment of the WG-DASL assay with respect to its applicability to signature validation and discovery. A study by Chien and colleagues [27] that identified differentially expressed genes associated with ovarian carcinogenesis using the WG-DASL assay demonstrated the potential of this assay for studying gene expression.

From a biological point of view, the stability of a particular mRNA is controlled by specific interactions between its structural elements and RNA-binding proteins [28]. External stimuli including temperature shifts and hypoxia, two conditions that arise when a sample undergoes the fixation process, can affect mRNA stability. From this perspective, a biological characterization (structural and functional) of transcripts that are accurately detected in FFPE as well as in FF samples, would add new insights into similarities and differences between FFPE and FF expression profiles.

In this paper we report an in-depth analysis of the WG-DASL assay on different aspects, in order to fully understand to which extent the DASL assay can be used with RNA from FFPE material for generating reproducible microarray data and signatures. We address this question in a four-step procedure. First, we focus on the reproducibility of FFPE gene expression profiles. Secondly, we analyze how comparable the gene expression profiles of matched FF and FFPE samples are. Next we apply three known gene expression signatures (Intrinsic subtypes, 70 gene prognosis signature and Genomic Grade Index), developed from FF samples, on FFPE samples in order to assess how well the FFPE material captures the biological information contained in these signatures. Lastly, we evaluate whether there are any specific biological characteristics that are correlated with the detectability of a transcripts extracted from FFPE material.

## Methods

### Patient samples

Twenty one breast cancer patients diagnosed in 2008 at the Netherland Cancer institute were selected for this study based on the availability of both FFPE (Formalin Fixed Paraffin Embedded) and FF (Fresh Frozen) material. For these patients, we collected 21 individual FFPE blocks and 21 individual snap frozen diagnostic biopsies. Sample characteristics are reported in the Table 1. Written informed consent was obtained from all patients included in the study. The study was approved by the ethics committee of the Netherlands Cancer Institute (NKI-AVL).

**Table 1:** Overview of the samples included in the study.

			FF_array_ID			FFPE_array_ID			RIN (FF)		PCR fragments (FFPE)		IHC		IHC/CISH		
pair_ID	FF_ID	FFPE_ID	a	b	c	a	b	c	a	b	a	b	ER	PgR	HER2	MP	grade
1	24720	2790	1	2	87	3	4	92	9.8	9.5	4	4	100%	0%	3	High	3
2	24926	4021	5	6	-	9	10	-	8.2	9.3	4	4	0%	0%	3	High	3
3	25287	5887	11	12	-	13	14	-	8.8	9	4	4	100%	10%	1	Low	1
4	25217	5536	15	16	-	17	18	-	8.5	8.8	4	4	80%	10%	3	High	2
5	25194	5371	19	20	-	21	22	-	8.9	8.3	4	4	100%	100%	0	Low	2
6	25418	6635	23	24	88	25	26	93	9.2	7.9	4	4	80%	80%	0	Low	1
7	25972	9001	27	28	89	29	30	94	8.1	9	4	4	100%	100%	0	High	2
8	24386	1053	31	32	-	33	34	-	9.3	8.5	4	4	70%	NA	1	High	3
9	24428	1221	35	36	-	37	38	-	9.6	9.3	4	4	100%	0%	3	High	2
10	24788	3264	41	42	-	43	44	-	8.9	8.9	4	4	0%	0%	1	High	3
11	24807	3428	45	46	90	47	48	95	9.2	9.1	4	4	75%	40%	0	High	3
12	25043	4732	49	50	-	51	52	-	7.4	8.3	4	4	1%	0%	3	High	3
13	25172	5290	53	54	-	55	56	-	8.6	8.2	4	4	100%	100%	3	High	2
14	25576	7296	57	58	-	59	60	-	7.9	8.2	4	4	100%	100%	0	Low	2
15	25468	6825	61	62	-	63	64	-	8.2	9.5	4	4	0%	0%	3	High	3
16	24266	0173	65	66	-	67	68	-	8.6	9.4	4	4	100%	80%	0	Low	1
17	24389	1070	69	70	91	73	74	96	8.9	9.5	4	4	100%	50%	1	High	2
18	25173	5292	75	76	-	77	78	-	7.9	7.6	4	4	100%	100%	1	Low	2
19	25298	5975	79	80	-	81	82	-	7.9	8.2	4	4	80%	0%	3	High	2
20	25575	7299	83	84	-	85	86	-	8	9.2	4	4	100%	60%	1	Low	1
21	25831	6473	not hybridized						4.9	5.9	4	4	0%	0%	3	NA	3

FF =  Fresh Frozen, FFPE =  Formalin Fixed Paraffin Embedded; pair_ID =  number of the pair FF-FFPE; FF (FFPE)_ID =  FF (FFPE) sample identifier; FF (FFPE)_array_ID =  sample identifier on the array; a, b =  biological replicates, c =  technical replicate; RIN =  RNA Integrity Number; ER =  Estrogen Receptor; PR =  Progesteron Receptor; HER2 =  Human Epidermal growth factor Receptor 2; IHC =  Immunohystochemistry; CISH =  Chromogenic In Situ Hybridization; MP =  MammaPrint, High =  high risk, Low =  low risk; grade =  histological grade; NA Not Available.

### RNA isolation

RNA from FFPE material was extracted using the High Pure RNA paraffin kit (Roche), which is the method recommended by Illumina for DASL applications [8], [9]. From the FFPE blocks 5 sections of 5 µm were cut and put onto a microscope slide (1 section/1 slide). A 4-µm pre-cut section was stained with haematoxylin and eosin and reviewed by a pathologist. Only blocks with ≥50% tumor cells were used. All the sections were micro-dissected by scratching off the enriched tumor cell area, using a sterile single-use scalpel and placed in a 1.5 ml reaction tube containing 1 ml xylene. The deparaffinization procedure was done in the tube. For each FFPE block two independent RNA isolations were performed, in order to have two biological replicates per sample.

RNA from FF material was extracted using the RNeasy Mini Kit (QIAGEN). Two 5-µm sections, which were cut before and after sectioning for RNA isolation, were stained with haematoxylin and eosin and reviewed by a pathologist to determine the tumor cell percentage. Only samples with ≥50% tumor cells were used. From the biopsy 15 sections of 30 µm were cut and placed in a 2 ml reaction tube containing 1 ml RNAzol B reagent (Biogenesis). The tissue was homogenized using a rotor-stator homogenizer and 200 µl of chloroform was added to the solution. After a centrifugation at 13000×g (4°C) for 15 minutes the upper aqueous phase containing RNA was transferred in a new vial. From this point on the manufacturer's protocol was followed, including an on-column DNase digestion for eliminating any DNA contamination. For each FF biopsy two independent RNA isolations were performed, in order to have two biological replicates per sample.

Depending on the tissue availability, we tried to match FFPE and FF tissues with similar tumor cell percentage, although differences of up to 10% in tumor cell percentage could be present.

The RNA concentration of the 84 samples (21 FFPE and 21 FF samples done in duplicates) was measured using the NanoDrop™ 2000 (Thermo scientific).

### RNA quality assessment

The RNA integrity of the FF samples was evaluated with the 2100 Bioanalyzer (Agilent) using the RNA 6000 Nano LabChip, following the manufacturer's protocol. The RNA Integrity Number (RIN) of the samples was above 8 in all cases except for one sample (in both replicates RIN<6), which was excluded from the subsequent analysis together with the FFPE counterpart.

The RNA integrity of the FFPE samples was determined by amplifying different length fragments (91, 123, 145 and 177 basepairs (bp)), of the glucose-6-phosphate dehydrogenase (G6PD) gene using the One-Step RT-PCR kit (Qiagen). The following 5′-3′ primers were used to amplify G6PD (RefSeq ID: NM_000402): F GAGGCCGTGTACACCAAGAT, R ATCTGTTGCCGTAGGTCAGG, F GCAACGAGCTGGTGATCC, R AGAAGACGTCCAGGATGAGG. Because the four oligonucleotides were added at the same time in the reaction mix, four combinations of forward and reverse primers were possible, allowing the amplification of four different length fragments. 300 ng of RNA was used as a template for reverse transcription (30 min at 50°C), followed by activation of the HotStarTaq polymerase (15 min at 95°C), 10 cycles of PCR (30 sec at 95°C, 30 sec at 68°C decreasing the temperature by one grade Celsius each cycle until reaching 58°C, 30 sec at 72°C), 30 cycles of PCR (30 sec at 94°C, 30 sec at 58°C, 30 sec at 72°C) and final 7 min extension at 72°C. PCR products were visualized on 2% agarose e-gels (Invitrogen). GeneRuler™ 100 bp DNA Ladder (Fermentas, Life Sciences) was used to determine product size. If, at the minimum, the PCR products of 123, 145 and 177 bp were visible on the gel, the quality of the RNA was considered acceptable for proceeding with the analysis.

The protocol above was developed in the diagnostic laboratory of the Pathology department at the Netherlands Cancer Institute and is routinely used for testing the quality of the RNA derived from FFPE material. In light of that, we opted for this approach, although Illumina would suggest a TaqMan Real Time PCR analysis for the FFPE RNA quality control [15].

### Whole Genome-DASL Assay

The Illumina Whole genome-DASL (cDNA-mediated Annealing, Selection, Extension and Ligation) assay was derived from the Human Cancer Panel DASL Assay [8], [9], [10], but differs from the original version by the number of transcripts assayed in parallel [2]. The assay measures 24526 annotated transcripts derived from the RefSeq database corresponding to 18391 unique genes. We performed the assay according to the manufacturer's instructions (WGDASL_Assay_Guide_11322443_B.pdf) by using approximately 100 ng of FF RNA and 200 ng of FFPE RNA. We assayed 96 samples in parallel: 45 FFPE samples (including 20 biological duplicates and 5 technical duplicates), 45 FF samples (including 20 biological duplicates and 5 technical duplicates), 1 FFPE pool RNA, 1 FF pool RNA in duplicate (a tumor reference pool described previously [17]), 1 commercial Total RNA extracted from HeLa cell lines (Invitrogen) in duplicate and 1 commercial Universal Human Reference RNA (Stratagene). The FFPE pool RNA was created pooling 100 ng of total RNA from each of the FFPE samples selected for the study. The labeled RNAs of 96 samples were hybridized to 12 HumanRef-8 Expression BeadChip arrays, each slide containing 8 identical microarrays. Microarrays were scanned using the Illumina Beadarray scanner, a confocal-type imaging system with 532 (Cy3) nm laser illumination. Image analysis and data quality assessment were performed using Genomestudio (Illumina). The average signal together with the 95^th^ percentile (p95) of the probe intensities on the array, were used for evaluating the quality of the hybridization. Because the p95 value represents a measure of the fluorescence intensity distribution of the data, a low p95 (p95<2500) corresponds to poor-quality hybridizations. Detection p-values were computed using several hundred negative controls to determine gene expression detection limits.

All the microarray data are MIAME compliant and have been submitted to ArrayExpress (E-TABM-1081).

### Data Analysis

Probes with an Illumina detection p-value below 0.05 in at least one of the samples (n = 90) (excluding FF pool, FFPE pool and control RNAs) were kept for the analysis, corresponding in total to 21205 probes. We assumed that a significant detection of a probe in at least one sample is sufficient to show the detectability of the probe in the DASL assay.

Data were normalized across the arrays using three different normalization methods available in the Genomestudio software package - simple scaling normalization (also called average normalization), cubic spline normalization and rank-invariant normalization (http://www.illumina.com/software/genomestudio_software.ilmn). For each normalization type we performed the calculation either with background subtraction or without background subtraction. We ran the analyses using all probes that passed the p-value filter or using a subset of them, termed the ‘informative probes’. For selecting the most informative probes we applied the following procedure: 1) we log2 transformed the normalized absolute intensity fluorescence value of each probe; 2) selected, for the FFPE and FF samples separately, the 20% highest variance probes, and 3) took the union of the two probe groups (FF and FFPE).

After the data normalization across arrays, we performed median centering on the probe log2 transformed intensities, considering FFPE and FF samples separately.

Next we calculated the Pearson correlation between the gene expression profiles (using all probes or the informative probes) of FFPE samples and their own replicates, as well as for FF samples and their replicates. We also measured the correlation between paired FFPE and FF samples. In order to show the specificity of the correlation between replicates of the same sample and between the FFPE/FF paired samples, we also report the average correlation of each sample against all other non-replicate/non-pair samples. The box plots (Figure S1) display the results obtained for each type of normalization, both with and without background subtraction (also referred as background correction). A box plot showing the number of probes with negative values following the background subtraction is also reported (Figure S2). Simple scaling normalization performed slightly better in the comparison between FF replicates, as well as in the comparison of FFPE samples against the FF paired samples and was the second best performing in the comparison between FFPE replicates (Figure S1). Therefore, since simple scaling is the most straightforward approach and performs well compared to rank-invariant or cubic spline normalizations, we selected simple scaling as our method to employ in all further analyses. Additionally, we opted for a normalization procedure without any background correction in order to avoid missing or negative values after logarithmic transformation of low intensities (Figure S2). As it is apparent from the box plots (Figure S1) FF Sample 69 is an outlier within the FF sample group using any normalization method. When we clustered all FFPE and FF samples using either all probes or the informative probes, with or without the median centering of the log2 transformed intensities, Sample 69 was never grouped with its biological replicate or with the FFPE paired samples (data not shown). Therefore, we decided to exclude this sample from the study.

After applying the p-value filter of the probes, excluding Sample 69, we ended up with 21178 probes (instead of 21205 probes including sample 69). The number of informative probes equals 5444.

Correlation analyses were performed in the statistical language R (http://www.r-project.org/). Heat maps were generated using the R statistics gplots package after simple scaling normalization and median centering of log2 transformed signal intensities on FFPE and FF samples separately. The distance between two samples in the heat map was calculated as one minus their Pearson correlation coefficient.

Unsupervised clustering analyses on FFPE samples were performed with BRB Array-Tools 3.8.1 (http://linus.nci.nih.gov/BRB-ArrayTools.html) and MeV 4.5.1 (http://www.tm4.org/mev/). Significance Analysis of Microarrays (SAM) [29] implemented in BRB Array-Tools 3.8.1 was used for the identification of differentially expressed genes. For the SAM analyses, 100 permutations were employed.

### Immunohistochemistry (IHC)

Immunohistochemistry for ERα (Estrogen Receptor alpha), PgR (Progesteron Receptor) and HER2 (Human Epidermal growth factor Receptor 2), and additional chromogenic in situ hybridization (CISH) for HER2 was performed and scored as described previously [30], [31]. In order to assess the correlation between immunohistochemical data of ERα, PgR and HER2 markers and microarray expression data of the corresponding genes, ESR1 (Estrogen Receptor 1), PgR and ERBB2 (v-ERB-B2 erythroblastic leukemia viral oncogene homolog 2, neuro/glioblastoma derived oncogene homolog (avian)) we used the Spearman's rank correlation (r_s_) [32] test implemented in SPSS Statistics Package 17.0. The absolute simple scaling-normalized intensities on the array for the genes ESR1 (Estrogen Receptor 1), PgR and ERBB2 were correlated with the percentage of IHC staining of ERα and PgR (ranging from 0% until 100%) and with the IHC-CISH status of HER2 (0, 1, 3). The size of the correlation was evaluated as follows: r_s_<0.33 small correlation, 0.33<r_s_<0.67 medium correlation, r_s_>0.67 large correlation.

### Intrinsic subtypes

We used the Single Sample Predictor (SSP) developed by Hu and colleagues [24] to define the molecular subtype of each sample. We mapped the Hu intrinsic gene list (306 genes) via Entrez ID and Gene Symbol ID to the WG DASL platform which resulted in 418 matching DASL probes (291 genes) that passed the p-value filter. When multiple probes were present for one gene, we selected the probe with the highest variance across the samples, ending up with 291 unique DASL probes. We calculated for all samples the Spearman correlation of a sample to the centroid of each molecular subtype. The predicted subclass of the sample was defined as the one with the highest correlation coefficient.

### Genomic Grade Index (GGI)

We mapped the 128 Affymetrix probes of the GGI signature [19] via RefSeq ID and Gene Symbol ID to the Whole Genome DASL platform and we obtained 141 DASL probes that passed the p-value filter (see Data Analysis for details). When there were multiple probes for one gene, we selected the probe with the highest variance across the samples, ending up with 110 unique DASL probes. We calculated the Genomic Grade Index (GGI) of all tumors as described in [19] where a sample was classified as high risk with a GGI ≥0 and as low risk with a GGI<0.

### 70-gene prognosis signature

We mapped the 70 genes previously reported [17], [33] via RefSeq ID and Gene Symbol ID to the Whole Genome DASL platform. Out of the 70 genes we found 60 genes on the DASL platform corresponding to 79 Illumina probes that passed the p-value filter (see Data Analysis for details). When multiple probes were present for a gene, we selected the probe with the highest variance across the samples. We calculated the Pearson correlation coefficient between the centroids of the original good prognosis template (as described in [17]) and the centroids of each sample with regard to the 60 genes. Tumor material of the 20 patients selected for this study had been analysed for MammaPrint (the commercial test of the 70 genes, marketed by Agendia, Amsterdam), and results of the test were available for this study.

### Biological feature identification of the DASL probes

For each probe that passed the p-value filter (n = 21178) the Pearson correlation coefficient between the log2 median centered intensities in the FFPE samples and in the FF counterparts (considering all replicates separately) was calculated. After ranking the probes based on their correlation value, we selected the top 1000 most highly correlating probes and the bottom 1000 least correlating probes. All calculations were conducted in the statistical language R. We extracted the following features for each transcript from the BioMart database (http://www.biomart.org/): transcript length, number of transcripts, probe start position measured from the 3 and 5 prime ends of the transcript respectively and transcript G/C content. We performed a Gene Ontology enrichment analysis using the DAVID Gene Functional Classification Tool 6.7 [34].

## Results

### RNA extraction and hybridization

Total RNA was successfully extracted in duplicate (biological replicates) from 20 Formalin Fixed Paraffin Embedded (FFPE) tissues and 20 matched Fresh Frozen (FF) tissues and hybridized to the DASL microarrays. Five FFPE samples and their FF counterparts were arrayed in duplicate (technical replicates). In summary 45 FFPE and 45 matched FF samples (Table 1) plus one FFPE pooled RNA, two FF pooled RNAs and three control RNAs (see Methods for details) were analysed on the Whole Genome DASL platform.

### Quality control

All 90 samples hybridized on the arrays passed the p95 and average signal quality control (see methods for details). FFPE samples had an average p95 signal equal to 11774 (±1111) and the FF samples had an average p95 signal equal to 13453 (±1047) (Figure S3 A). FFPE average signal intensity was 3062 (±366) and FF average signal intensity 3936 (±372). In terms of average p95 signal and average intensity the two groups (FFPE and FF) were significantly different (Mann-Whitney U test p-value<0.001). While the signal intensity of the FFPE samples was consistently lower it was still well above the quality control threshold (p95>2500) in all cases.

When considering the raw log2 transformed intensities, FFPE samples showed a comparable distribution of signal intensities compared to the FF samples although the FFPE intensities were slightly lower on average, as was seen in the p95 analysis.

The average number of probes detected above background (p-value <0.05) in FFPE samples was comparable with the number of probes detected in the matched FF samples (16518±718 in FFPE versus 17418±462 in FF samples) although lower on average (Mann-Whitney U test p-value<0.001) (Figure S3 B). Of the 16518 (±718) probes, 11883 probes (approximately 72%) were detected in all FFPE samples (n = 45), a slightly higher percentage was detected in all the FF samples (n = 45) (77%, 13370/17418 common probes). Of the 11883 probes that were detected in all the FFPE samples, 11139 (94%) overlapped with the 13370 FF probes detected in all samples. Three pairs (pair_ID = 10, 15, 17) showed a bigger difference in the number of detected probes between matched FFPE and FF samples in respect to the other pairs, as can be seen from the Figure S3 B).

### Reproducibility of the Whole Genome DASL assay

**Figure 1:**
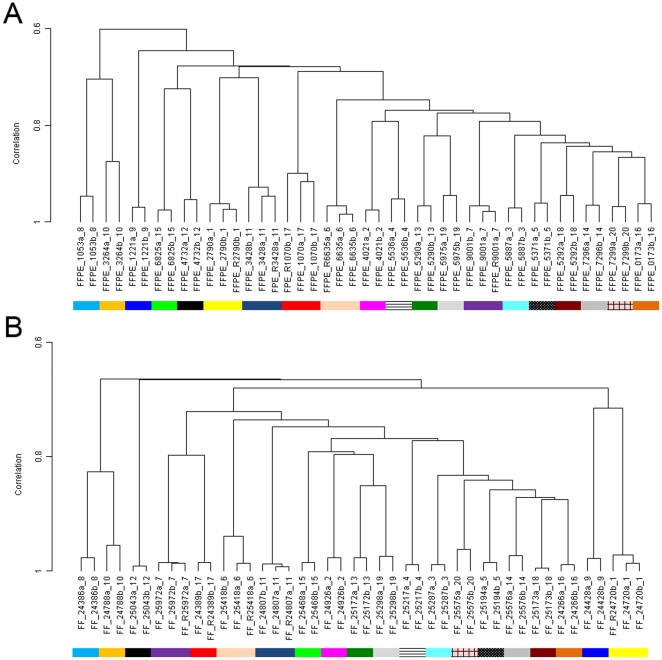
Unsupervised hierarchical clustering with the most informative 5444 probes of all FFPE samples (n = 45) (A) and all FF samples (n = 44) (B). In both cases (A and B) the samples cluster together with their biological and technical (if present) replicates. We employed simple scaling normalization without background subtraction and log2 transformed the data. The replicates are color coded. On the left side of the dendogram is reported the Pearson correlation (from 0.6 to 1).The samples are indicated with the FFPE_ID or FF_ID and the Pair_ID combined. FFPE = Formalin Fixed Paraffin Embedded, FF = Fresh Frozen.

To assess the reproducibility of the WG-DASL assay for FFPE samples, we evaluated the gene expression profiles of 45 FFPE samples which included 15 samples in duplicate (two biological replicates) and five samples in triplicate (two biological replicates and one technical replicate). Unsupervised hierarchical clustering of the FFPE samples only, with the 5444 most informative probes showed that, as expected, all replicates clustered together, (Figure 1A), showing a high level of reproducibility for the FFPE protocol. The average Pearson correlation between replicates was 0.95 (±0.02). When we repeated the clustering analysis using all probes (n = 21178) the average correlation between replicates was 0.98 (±0.01) (data not shown). Although the correlation between replicates was slightly lower when only the most informative probes were used, the correlation between the samples became more specific for sample pairs. When we clustered the FFPE samples using the 5444 most informative probes the correlation between non-replicate samples changed from 0.91 (using all 21178 probes) to 0.69. Interestingly, replicates of FF samples (n = 44) showed similar results with an average Pearson correlation coefficient of 0.99 (±0.01) on all probes and 0.98 (±0.01) on the most informative probes. (Figure 1B).

### Comparison between gene expression profiles of FFPE and FF paired samples

In order to assess the performance of the WG-DASL assay we compared the gene expression profiles of the 20 matched FFPE and FF pairs.

We used simple scaling normalized and median centered log2 transformed intensities. The reason for median centering the data is related to the difference in tissue types we are comparing. Besides an overall bias in the signal intensity, which is corrected for by the simple scaling normalization procedure, additional probe specific bias may exist. By median centering each probe separately for each group (i.e. FF and FFPE), part of this bias can be eliminated. In effect, the median intensity value of each probe in each group is shifted towards zero which corrects for consistently higher or lower signal in either group. A similar strategy has been used previously in a different context, to adjust for platform bias [35], [36].

**Figure 2:**
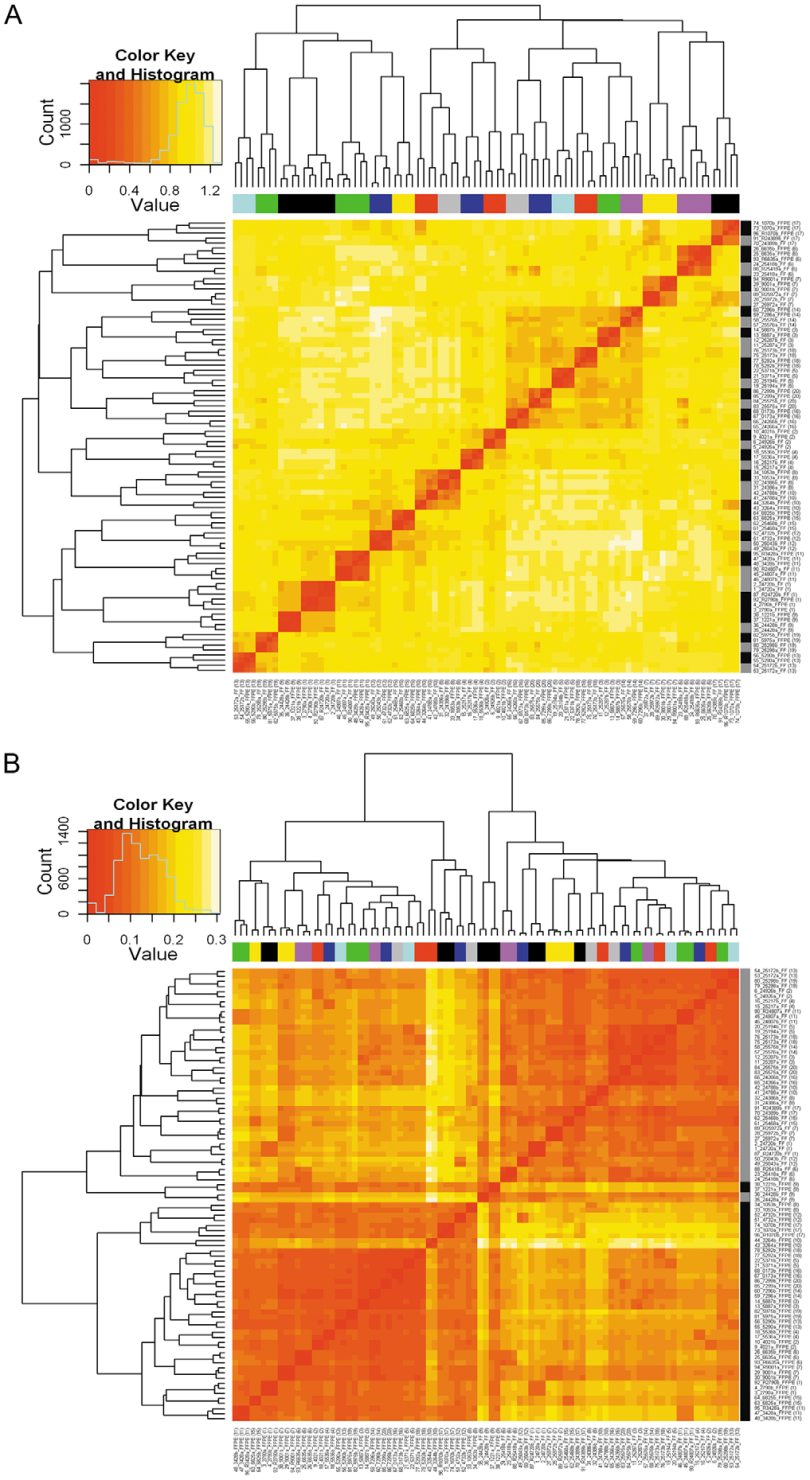
Heat maps of the distance matrix representing the pairwise distances between all FFPE and FF samples (n = 89) using all probes that passed the p-value filter (n = 21178). Prior to the computation of the distance matrix (where distance = 1-Pearson correlation), data were normalized with a simple scaling normalization without background correction and then median centered per probe, separately for FFPE and FF samples. The paired samples are indicated with a number from 1 to 20 and are color coded. The tissue type is indicated with the vertical bar (black = FFPE, white = FF). (**A**) Heat map using median centered log2 normalized data. (**B**) Heat map using non-median centered log2 normalized data.

The heatmap of the distance measures of all FFPE and FF samples (n = 89) was generated using all probes that passed the p-value filter (n = 21178) (Figure 2A). In this analysis all FFPE and FF pairs clustered together. Importantly, when we did not median center the data, the FFPE and FF paired samples did not cluster together, rather, all FFPE tissues and all FF tissues clustered together and the replicates within these separate groups clustered together (Figure 2B). We obtained similar results using the most informative probes (n = 5444) (Figure S4).

**Figure 3:**
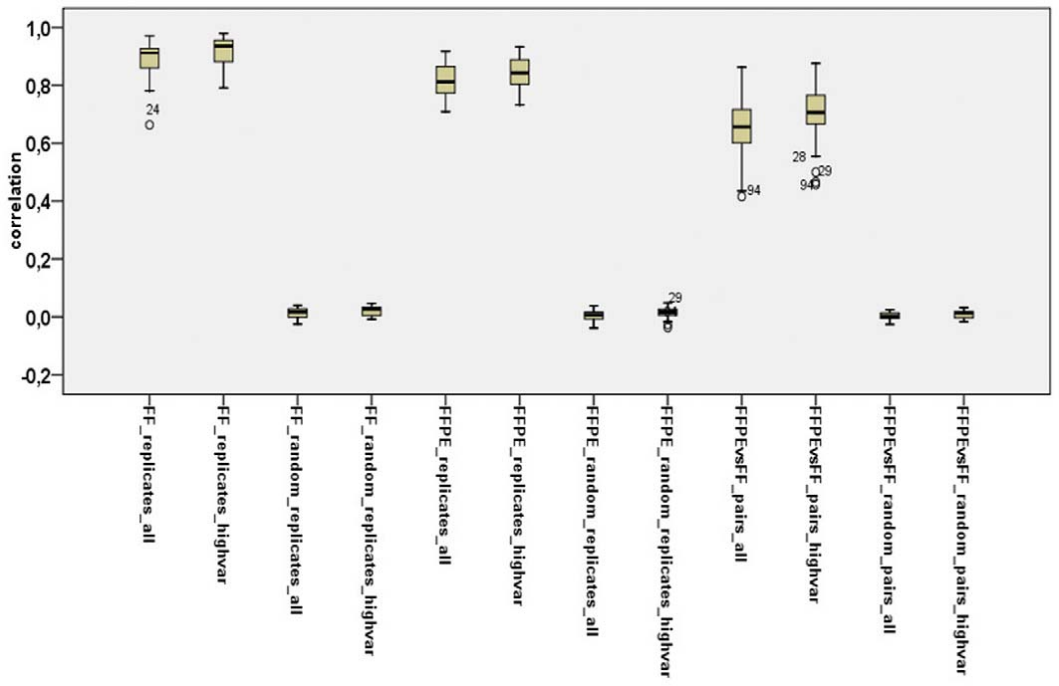
Box plots of the Pearson correlation coefficient between FF replicates (n = 44), FFPE replicates (n = 45) and FFPE/FF pairs (n = 44). The box plots were generated using all probes that passed the p-value filtering (all) or using the informative probes (highvar). The data were normalized across the arrays with a simple scaling normalization and median centered per probe. The outlier samples are indicated in the graph with the array_ID (see Table 1). FF_replicates =  FF replicates, FF_random =  FF random replicates, FFPE_replicates =  FFPE replicates, FFPE_random =  FFPE random replicates, FFPEvsFF_pairs =  FFPE-FF paired samples, FFPE vs FF_random_pairs =  random FFPE-FF pairs.

As can be seen in Figure 3, the distribution of Pearson correlation coefficients generated from the comparison between FFPE and FF paired samples differed significantly from the distribution of the randomly paired samples (non pairs). For FFPE and FF paired samples the average Pearson correlation was 0.65 (±0.11) using all probes and 0.70 (±0.10) using only the most informative probes. For randomly paired samples the Pearson correlation coefficient decreased to values around zero with all probes (r = 0.002±0.01) and with the most informative probes (r = 0.009±0.01). When we used normalized non-median centered data, the average correlation between FFPE and FF pairs increased from 0.65 to 0.89 (±0.03) using all probes and from 0.70 to 0.80 (±0.05) with the most informative probes. However, at the same time, the non-pair correlation increased from 0.002 to 0.83 (±0.02) with all probes and from 0.009 to 0.59 (±0.05) with the most informative probes, overall lowering the difference in correlation between pairs and non-pairs. As expected within samples of the same tissue type the Pearson correlation coefficient between biological replicates is higher (>0.8 in FFPE and FF samples) than the one between FFPE and FF paired samples (Figure 3).

**Figure 4:**
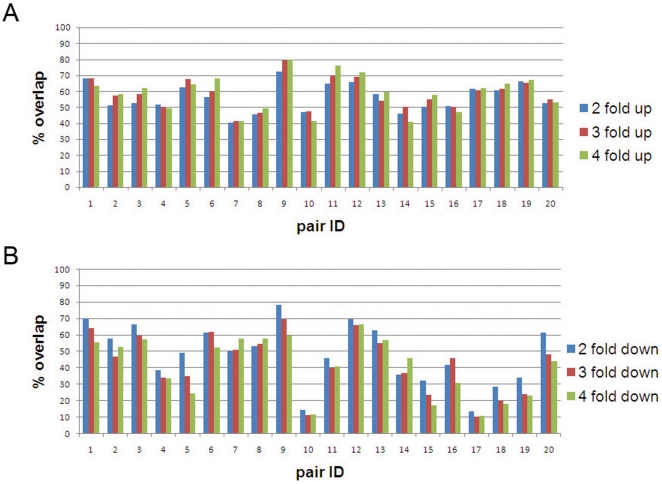
Percentage overlap between the two-, three- and four-fold regulated probes in FFPE and FF paired samples for the up-regulated probes (A) and down-regulated probes (B).

Next we performed differential gene expression analysis in order to determine whether the results generated with FFPE expression data were similar to the ones obtained with FF expression data. First we identified the two- three- and four-fold differentially expressed probes (of the 21178 p-value selected probes) in FFPE and FF paired samples. Fold difference was computed with respect to the average signal of all the samples from the same tissue-type. Signal intensities of the two biological replicates of each sample were averaged. The percentage overlap between FFPE and FF paired samples of two-, three- and four- fold up-regulated probes was similar, in contrast to the number of down-regulated probes that showed more variability (Figure 4). The average percentage overlap between the two- three- and four- fold up-regulated probes in FFPE and in the corresponding FF paired sample was 56%, 58% and 59% respectively. For the two- three- and four-fold down-regulated probes the percentage overlap decreased to 48%, 43% and 41% respectively. We observed that in the majority of the pairs (15/20) there was an increase in common, overexpressed probes between FFPE and FF matched samples when going from two-fold to four-fold change. On the other hand, the number of common, under-expressed probes between FFPE and FF pairs tended to decrease when going from two-fold to four-fold change in most of the pairs (17/20). We observed that the pairs with IDs 10 and 17 showed an overlap below 15% in the underexpressed probes, a result that could partly be explained by the low Pearson correlation previously observed for these two pairs (r<0.5). These results are also in concordance with the low overlap in the number of significantly detected probes found for the pairs 10 and 17 and previously reported (Quality control section). A possible reason of the low overlap could be a difference in term of tumor cell percentage between the FFPE tissue and its FF counterpart. Indeed, pair number 10 showed a tumor cell percentage of 70% in the FFPE tissue block and of 60% in the FF biopsy. Also, for the pair 17 we observed different tumor cell percentages, although in both types of specimen the percentage was high (90% FF and 80% FFPE).

As a representation of common group versus group differential gene expression analysis we subsequently identified differentially expressed probes between histological grade 1 (n = 9) and histological grade 3 (n = 16) tumors (see Table 1) for both FFPE and FF paired samples using SAM (Significance Analysis of Microarray) [29]. With a False Discovery Rate (FDR) set to ≤0.01 and a fold-change ≥2, approximately 53% (718/1350) of the probes identified in the FFPE sample comparison overlapped with those identified in the FF paired sample comparison. This degree of overlap was found to be significant (Hypergeometric test p-value <0.001). Interestingly when we considered higher fold changes (from 5 until 8) we observed an increase in the overlap ranging from 60% up to 81% (data not shown). This result is consistent with data previously reported using a different assay [13].

### Correlation between microarray data and Immunohystochemistry (IHC)

In order to evaluate if the FFPE microarray data correlated with the immunohistochemical data for the single gene markers ERα (Estrogen Receptor alpha), PgR (Progesteron Receptor) and HER2 (Human Epidermal growth factor Receptor 2) we recovered the absolute normalized intensities of the corresponding genes, ESR1, PGR and ERBB2 from the array for the FFPE samples (n = 45). Three probes represent ERBB2 on the array; there is one probe specific for ESR1 and one for PGR.

The ESR1 array intensity showed a significant Spearman rank correlation with the ERα IHC percentage (r = 0.71, p-value <0.01), the PGR array intensity also showed a significant correlation with the PgR IHC percentage (r = 0.89, p-value <0.01). All three probes specific for ERBB2 significantly correlated with the HER2 CISH-IHC status although two of them showed a higher correlation coefficient (r_1_ = 0.82, p-value <0.01; r_2_ = 0.57, p-value <0.01; r_3_ = 0.81, p-value <0.01) (Figure S5).

### Reproducibility of gene expression signatures on FFPE samples

We applied three gene known expression signatures developed from fresh frozen samples (the Hu molecular subtype signature [24], the Genomic Grade Index [19] and the 70 gene prognosis signature [17]) to our samples in order to determine whether FFPE and FF matched pairs agreed in terms of their class predictions.

Table 2A reports the predicted molecular subtypes of all FFPE and FF matched samples using the intrinsic subtype Single Sample Predictor (SSP) developed by Hu and colleagues [24]. This prediction was based on the 291 DASL genes that mapped to the original intrinsic gene list. Of the 20 FFPE and FF pairs, 19 showed an agreement in terms of molecular subtype prediction across all replicates. Only in one case we found that the FFPE biological replicates were classified as normal-like and the FF replicates as luminal A (pair_ID = 2). This conflicting result could be due to within-tumor heterogeneity between the FFPE and the FF tumor samples used in the analysis as observed by others in a similar analysis [16]. When we looked at the correlation values of the discordant replicates to the subtype centroids, the FFPE replicates showed a Spearman coefficient to the normal-like and the luminal A centroids that differed by less than 0.01 (data not shown). Given the fact that the majority of the patients are hormonal receptor positive (Table 1), a high number of luminal type tumors is expected. Nevertheless, in few cases a luminal A subtype was predicted where the tumor had an ER negative status (pair ID = 15 in both FFPE and FF replicates, pair ID = 2 in FF replicates). In concordance with IHC assessment the expression levels of the ESR1 gene for these samples was the lowest among the samples predicted to be of the luminal subtype (the level of expression was similar to the non-luminal type level of expression). This implies that the disagreement between IHC status and subtype is influenced by the expression levels of other probes used for the classification.

**Table 2:** (**A**) Hu Intrinsic subtype prediction for FFPE and FF matching pairs (n = 20). (**B**) Genomic Grade Index (GGI) prediction for FFPE and FF matching pairs (n = 20).

A			
Subtype	FFPE	FF	Concordance a
Luminal A	16 (16/16)^b^	17 (17/17)^b^	16/17
HER2	1 (1/1)^b^	1 (1/1)^b^	1/1
Basal	2 (2/2)^b^	2 (2/2)^b^	2/2
Normal	1 (1/1)^b^	0	0
tot	20	20	19/20

B			
GGI	FFPE	FF	Concordance a
High GGI	10 (8/10)^b^	9 (9/9)^b^	9/9
Low GGI	10 (10/10)^b^	11 (10/10)^b^	10/11
tot	20	20	19/20

a Number of FFPE pairs concordant with the FF matched pairs.

b Number of concordant replicates of the same tissue type.

Next we classified the samples in high risk or low risk based on the Genomic Grade Index (GGI), calculated as reported in [19], using 110 DASL probes matching the original GGI signature. The results are summarized in the Table 2B. Of the 20 FFPE/FF pairs, 19 had the same risk prediction in the FFPE and FF matching pairs. In one of the 20 pairs (pair_ID = 7), there was a different prediction in all FFPE replicates (low GGI risk) compared to all the FF replicates (high GGI risk). Heterogeneity between the FFPE and the FF tumor samples as previously observed for the intrinsic subtype analysis, could explain this conflicting result.

When we performed an unsupervised hierarchical clustering (HCL) on all samples with the 110 DASL probes, the samples grouped in two main clusters, enriched in low GGI risk and high GGI risk samples (Figure S6). Although in three cases we observed discordant GGI results within biological replicates of the same tissue type (pair ID = 13, 17, 19), the discordant samples did cluster with the other replicates.

When we compared the GGI classification with the histological grade we found on average a good concordance, in agreement with the results from the clustering analysis (Figure S6). All four grade-1 tumors were predicted correctly to be low GGI risk. Six of the seven grade-3 tumors were classified correctly as high GGI risk and only one (pair ID = 2) as low GGI risk. We observed a lower concordance in the GGI prediction for the nine histological grade -2 tumors. Five were concordantly classified as high or low risk GGI in FFPE and FF matched samples. Three showed a discordant result between replicates of the same tissue type (pair_ID = 13, 17, 19) and one showed discordance between the FFPE sample and the FF matched pair (pair_ID = 7).

**Figure 5:**
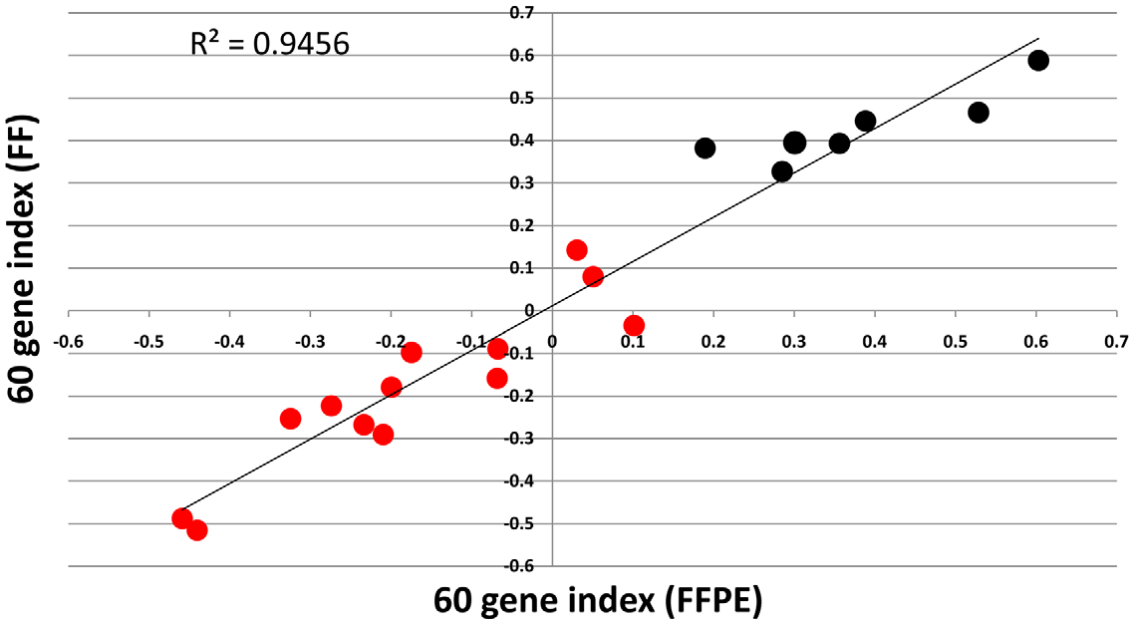
Comparison of the 60 gene index as derivative of the 70 gene prognosis signature of the FFPE samples to the 60 gene index of the paired FF samples. Scatterplot of the Pearson correlation of the 60 genes from each sample to the average expression profile of the good outcome patients as described in [17], [33]. This correlation is defined as the 60 gene index. The circles are colored based on the diagnostic results for these patients of the customized MammaPrint 8-pack test [33]: red = high risk outcome, black = low risk outcome.

Lastly we calculated a 60-gene index as derivative of the 70 gene prognosis signature for the FFPE and FF pairs. The 60-gene index represents the 60 genes matching the 70 genes previously reported [17], [18]. By computing the Pearson correlation coefficient of the expression values of these genes and the centroids of the original good prognosis template [17], a prognostic class can be assigned to each sample. A scatterplot of the 60-gene index of the 20 FFPE samples against the 60-gene index of the 20 FF paired samples is shown in Figure 5. The r-squared measure of the linear regression of the data is 0.94, indicating a high concordance between FFPE and FF samples. The 60 gene index correlated very well with the results of the MammaPrint diagnostic test [33] as emerged when we calculate the Pearson correlation coefficient between the reported MammaPrint indices and the FFPE or FF 60-gene indices (r = 0.88 and r = 0.89 respectively). Taking into account that the MammaPrint and the 60 gene index are obtained from different microarray system, this level of concordance shows the potential inter-platform reproducibility of the 70 gene profile. However, a larger study will be required to determine an optimal threshold on the 60 gene index for separating high-risk from low-risk patients.

### Biological investigation of the DASL data

**Figure 6:**
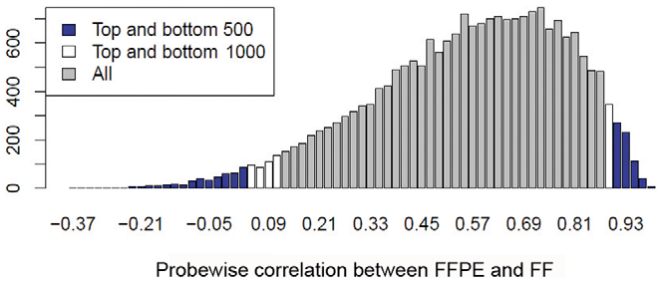
Distribution of the probewise correlation between FFPE and FF matching pairs. The x-axis reports the probewise correlation between FFPE and FF matching pairs, the y-axis the corresponding number of probes. The 1000 and 500 most and least correlating probes are highlighted in white and blue respectively.

The 1000 probes showing the highest (top 1000) and lowest (bottom 1000) correlation between FF and FFPE samples were identified as described in the Methods. Figure 6 shows the distribution of the Pearson correlation coefficient of all probes (n = 21178). Interestingly, 72% of the top 1000 correlating probes overlapped with the 5444 informative probes selected on the basis of the variance across the samples. In contrast, only 11% of the least correlating 1000 probes were included in the informative probe list. This is to be expected because the most informative probes are less prone to technical noise and therefore showed the highest concordance between the two tissue types.

First we evaluated if there was a tendency for highly correlating probes to also be high intensity probes. As can be seen in Figure S7 there is no association between the level of correlation and the intensity of the probe. The high density regions (darker areas in Figure S7) can be explained by the fact that the majority of the 21178 probes show a correlation between 0.5 and 0.8 (Figure 6, Figure S8). The top correlating probes have an intensity distribution resembling that of probes across the whole range of observed correlation coefficients. (Figure S9).

**Figure 7:**
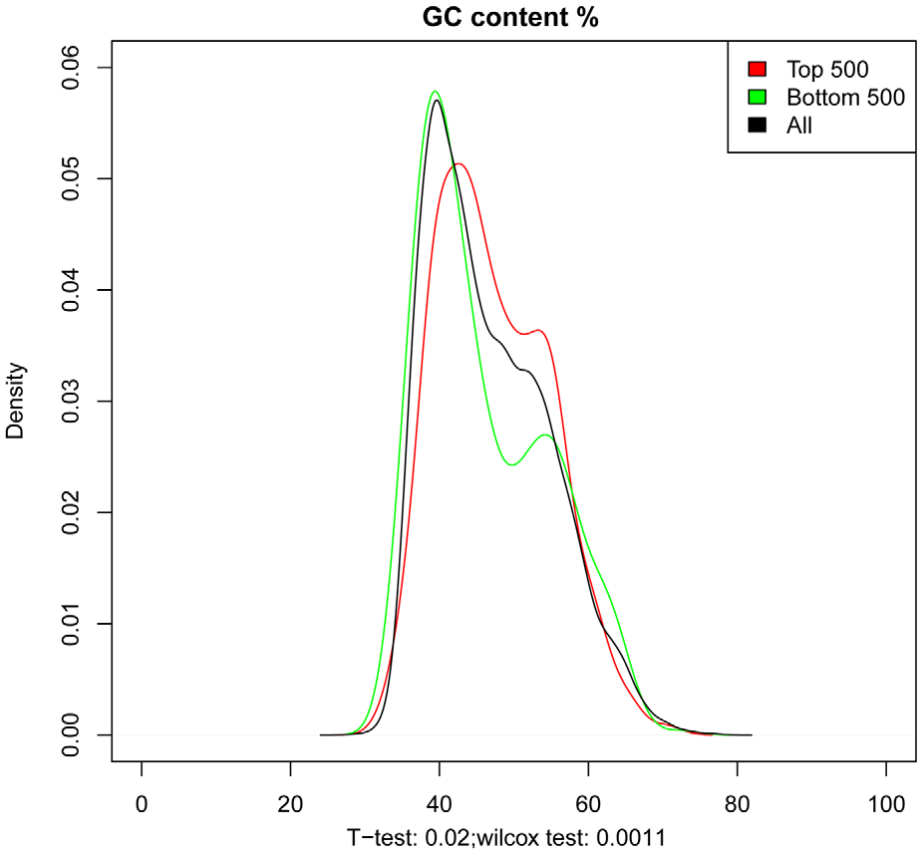
Density distribution of the GC content (%) in the top 500 most highly correlating and bottom 500 least correlating probes. Below the x axis the statistical significance based on the T-test and Wilcoxon signed-rank test is shown. Similar density functions were observed for the top 1000 and bottom 1000 probes.

In order to identify any specific biological characteristics associated with the most and least correlating probes, we evaluated different features of the corresponding transcripts. We evaluated the following features: 1) probe start position from the 3 prime or 5 prime ends of the transcript; 2) transcript length; 3) number of transcripts associated with the probe and G/C content. G/C content showed the strongest difference between the top 1000 (or 500) and bottom 1000 (or 500) probes (Figure 7). Interestingly, the transcripts show a higher correlation between FFPE and FF paired samples, had a higher G/C content percentage than the less correlating ones.

Since better correlating transcripts between FFPE and FF matching samples are likely less affected by the degradation, we wanted to evaluate if a difference in detectability could be related to the biological function of the transcript. Therefore, we performed a Gene Ontology (GO) analysis to determine whether the top and bottom correlating transcripts differed in terms of their functional annotation as represented by the three classes in the Gene Ontology: biological process, molecular function or cellular component categories. The top 1000 probes showed enrichment in biological categories mainly related to the cell cycle such as cell cycle phase, M phase, mitosis, nuclear division. The bottom 1000 probes revealed, on the other hand, a broader spectrum of biological categories: DNA repair (regulation of DNA repair, mismatch repair, response to DNA damage stimulus), RNA processing (RNA splicing, spliceosoma, mRNA processing, ribonucleoprotein complex) or transport (intracellular transport, protein transport).

## Discussion

Gene expression profiling of FFPE samples represents great potential for translational cancer research. Unfortunately this type of analysis has proven to be problematic because of the poor quality of RNA extracted from FFPE. In this study we showed that expression data with sufficient quality for further genomic analysis can be generated from FFPE material using a whole genome expression assay specifically designed for degraded RNA, the DASL Whole Genome assay.

In our analysis we selected 20 FFPE blocks from 2008 from a single pathology department, in order to reduce the chance of failure due to the age of the tissue block or differing tissue processing methods. Block age, RNA extraction method and optimal concentration for a successful DASL assay, were previously evaluated [8], [10], [16], [37] and are aspects beyond the scope of this study.

There is a high reproducibility of FFPE gene expression profiles using 20 biological and technical replicates derived from the FFPE samples as was evident from unsupervised cluster analysis. The Pearson correlation between biological replicates was greater than 0.90, comparable to the correlation coefficient previously shown for technical replicates [16]. Importantly, as additional sign of reproducibility of the DASL assay on FFPE material, we found that the average correlation coefficient between FFPE replicates was similar to the one obtained with FF replicates.

DASL array data and Immunohistochemical data of prognostic markers used in the clinical setting (ER, PgR, HER2) significantly correlated with the corresponding gene expression values when we considered the FFPE samples.

Different approaches used for comparing matched FFPE and FF tissues showed on average a good degree of comparability. Direct comparisons of paired FFPE and FF gene expression profiles considering a subset of most informative probes and using normalized, median centered data, yielded an average Pearson correlation of 0.7, in accordance with what was previously reported with the same assay [2]. Other studies with a different assay system [7], [13] reported higher correlation coefficients between FFPE and FF matched samples (r>0.7) using raw or normalized data, but without median centering the probe intensities. Indeed we observed that with non-median centered data the average correlation between FFPE and FF pairs increased from 0.70 to 0.80, but at the same time also the non-pair correlation raised from 0.009 to 0.59. A possible explanation for this phenomenon can be found in the fact that genes that are highly expressed in all samples will lead to a higher correlation between samples (regardless of whether the samples are paired or not). By median centering, the expression values of these genes are shifted towards zero, thereby lowering the correlation coefficient. This explains the lower correlation scores observed both in non-paired samples and in paired samples. We concluded that the discriminatory power between pairs and non-pairs is more important than absolute correlation values. Probe based median centering normalization on FFPE and FF samples processed separately proved to be an excellent way of data normalization in our study and eliminated the systemic technical differences in signal intensity between FFPE and FF paired samples. Advantages in processing FFPE and FF assays separately were also observed by others [13]. The dramatic effect of the median centering procedure is evident when we look at the heatmap generated with (Figure 2A) and without (Figure 2B) median centering the data. If we do not center the data FFPE and FF matched samples group completely separately in an FF and an FFPE cluster, in contrast to median centered data where all the FFPE samples clustered together with their FF paired counterparts.

We observed a good overlap (>50% relative to the FFPE samples) between differentially expressed genes detected in FFPE and FF pairs, using the histological grade (grade 1 versus grade 3) as the class label and this overlap tended to increase when considering higher fold changes (from two until eight fold). This finding is in accordance with previously reported results with a different assay [13] where normal tissue was compared to tumor tissue. A similar comparison was performed by Illumina with the WG DASL assay and in this case they showed a higher percentage of overlap between differentially expressed gene lists (>70%). However, it should be taken into account that in that study normal tissue was compared against tumor tissue and differences between these two tissue types are more pronounced [2]. When calculating the number of common 2-, 3- and 4-fold differentially expressed probes between FFPE and FF samples, relative to the average signal of all FFPE or FF samples, the average percentage overlap was smaller, especially for the underexpressed probes. This discrepancy could be due to the fact that in the first analysis we made a biological contrast (the histological grade) between groups of FFPE and FF pairs, a contrast that was missing in the fold change analysis where individual FFPE or FF samples were compared to the average signal. In a “real life” experimental set up, the first approach described would be the most probable to be used for identifying differentially expressed genes.

With the application of three known gene signatures developed from fresh frozen material on our FFPE series using proper adjustments, we showed that it is possible to extract meaningful biological information from partially degraded samples, with respect to fresh frozen intact samples treated as reference. All FFPE and FF samples, with the exception of one case, were predicted to have the same molecular subtypes. The majority of the pairs were classified as either high risk or low risk over all FFPE and FF replicates using the DASL GGI signature. Although some discordance was observed when we applied the DASL GGI classifier, it is interesting to that, except for one case, all samples clustered into two main clusters, in accordance with their expression profile risk.

In the 70-gene analysis the degree of concordance between matched FFPE and FF samples was high (R^2^ = 0.94) and, most intriguing, the prognosis prediction made using 60 DASL gene index showed a trend in agreement with the prediction of the diagnostic test MammaPrint [33]. MammaPrint is a customized microarray based on the Agilent technology that represents a completely different platform compared to the DASL Illumina assay we used. These data, besides demonstrating the robustness of the DASL assay, open up new possibilities for analyzing gene expression using microarray- based assays on FFPE material.

The degree of concordance between FFPE and FF pairs with respect to three known gene signatures demonstrates that an overlap below 70% between differentially regulated genes in FFPE and FF pairs is sufficient to produce highly similar predictors for breast cancer. By definition FFPE and FF samples cannot be exactly the same as these are typically sampled from a different part of the tumor). In addition, most likely, real differences exist between FFPE and FF results and these differences are due to the different protocols used. However, expression profiles are highly consistent when using the same protocol (>0.9 Pearson correlation). Because of this high within-protocol consistency, FFPE material could be used to discover new signatures and these could readily be applied to new FFPE samples. What this study shows is that using proper normalization methods, signatures derived from FF material with sufficient signal can be accurately translated to an FFPE derived signature. This implies that “the prognostic signal” is present in both the FF and the FFPE material and as such, the reverse must also be true. That is, a signature discovered in FFPE material can be translated to its FF counterpart when the appropriate normalization steps are taken into account. This opens up great opportunities given the large amounts of FFPE stored material and the associated clinical data.

When we investigated the biological characteristics associated with the top and bottom correlating transcripts (between FFPE and FF matched samples) the G/C content showed to be higher in the top correlating genes. Our finding could suggest that a higher GC content is associated with a better detectability in FFPE material and therefore to mRNA stability. Although this remains a hypothesis, it seems to be consistent with what previously observed that most of the mRNAs that harbor coding region instability elements happen to be GC-poor [38].

Interestingly the top correlating transcripts showed enrichment in Gene Ontology biological categories related to cell cycle processes. Considering the idea proposed in [28] that translational repression during mitosis would inactivate mRNA decay pathways stabilizing many labile mRNAs, we could hypothesize that genes involved in the mitosis process are less affected by degradation (induced by the formalin paraffin-embedded procedure) and therefore better correlating with the genes expressed in more intact tissues, such as FF material.

Taken together the results of this investigation demonstrate that the DASL assay, which was specifically designed for partially degraded RNA from FFPE material, combined with a proper way of data processing, allows one to obtain reproducible and usable expression data from FFPE material. While the proposed normalization procedures compensate, for a large part, for the systematic bias between FFPE and FF material, a biologically induced difference between the two is evident. Particularly the function and the GC-content of a particular gene appear to play a role in the stability of the mRNA of that gene in FFPE material. Future studies making use of FFPE material should carefully consider these factors and take great care to apply proper normalization procedures as this can have a big impact on the results. FFPE gene expression data contains biological information comparable to the data generated from the intact counterpart that make them a valuable and useful resource for prognostic risk assessment and other genome wide analyses.

## Supporting Information

Figure S1 **Box plots of the Pearson correlation coefficient between FF replicates (A), FFPE replicates (B) and FFPE/FF pairs (C) using three normalization procedures: simple scaling (ss), rank-invariant (ri) and cubic spline (cs) and carried out for either with background correction (bgc) or without background correction (nobgc).** The box plots were generated using all probes that passed the p-value filtering (all) or using the informative probes (highvar). The number of informative probes using ss normalization without bgc is equal to 5480, with bgc is equal to 5284. The number of informative probes using ri normalization without bgc is equal to 6351, with bgc is equal to 6323. The number of informative probes using cs normalization without bgc is equal to 5388, with bgc is equal to 5664. In Panels A, B we report the correlation distribution of the comparison between real replicates (replicates) and between non-replicate samples (random_replicates). In Panel C we report the correlation distribution of the comparison between real pairs (pairs) and between non-paired samples (random_pairs). The circles in the graph are outlier samples indicated with the array_ID (see Table 1). The legend on the horizontal axis is a concatenation of the abbreviations for the normalization, sample pairing, probe set employed and the type of background correction applied.
(TIF)

Figure S2 **Number of probes on the array with negative values (na) after application of different normalization methods.** The x-axis displays the normalization method (ss = simple scaling, ri = rank invariant, cs =  cubic spline, nobgc =  without background subtraction, withbgc =  with background subtraction) and the dataset used (all =  all probes that passed the p-value filtering, highvar =  only informative probes). The y-axis represents the number of probes. The box plots represent the distribution of the number of probes with negative values for each option evaluated. If present, outlier samples are indicated with a black circle. The total number of probes in the dataset is represented by the blue circles.
(TIF)

Figure S3 **Quality control of the DASL gene expression data.** (**A**) 95^th^ percentile of the fluorescence intensity in FFPE and FF paired samples. On the x-axis we report the Pair_ID (1-20) and on the y-axis the 95^th^ percentile (P95) of the fluorescence intensity. (**B**) Number of detected probes above background in FFPE and FF paired samples. On the x-axis we report the Pair_ID (1-20) and on the y-axis the number of probes significantly detected above background (p-value>0.05).
(TIF)

Figure S4 **Heat maps of the distance measures (1-Pearson correlation coefficient) of the FFPE and FF samples (n = 89) using the informative probes (n = 5444).** (**A**) Heat map using median centered log2 normalized data. Displayed are the distance measures of the FFPE and FF samples using all probes for calculating the distance sample by sample. Data are normalized with a simple scaling normalization without background correction and then median centered per probe, separately for FFPE and FF samples. Distances range from 0 (minimum distance) to 1.2 (maximum distance) as shown in the top left panel. The paired samples are indicated with a number from 1 to 20 and are color coded. (**B**) Heat map using non-median centered log2 normalized data. Displayed are the distance measures of the FFPE and FF samples using 5444 for calculating the distance sample by sample. Data are normalized with a simple scaling normalization without background correction. Distances range from 0 (minimum distance) to 0.6 (maximum distance) as shown in the top left panel.
(TIF)

Figure S5 **Comparison between the Immunohistochemistry (IHC) marker status (ER, PgR, HER2) and the gene intensity on the array (ESR1, PGR, ERBB2) for the FFPE samples (n = 45).** (**A**). Dot plot of ER/ESR1: The x-axis reports the IHC percentage of staining of ER; the y-axis reports the absolute intensity of the gene ESR1 on the array. (**B**) Dot plot of PgR/PGR**:** The x-axis reports the IHC percentage of staining of PgR; the y-axis reports the absolute intensity of the gene PGR on the array. (**C**) Dot plots of HER2/ERBB2: The x-axis reports the IHC-CISH (Chromogenic In Situ Hybridization) status of HER2; the y-axis reports the absolute intensity of the gene ERBB2 represented by three different probes on the array (**I**, **II**, **III**).
(TIF)

Figure S6 **Unsupervised hierarchical clustering of all FFPE and FF samples (n = 89) with 110 DASL probes matching with the Genomic Grade Index (GGI) gene list.** The clustering is performed using Pearson correlation and average linkage. The paired samples are color coded (pair_ID). Samples were classified as low risk or high risk based on the GGI score (see Methods for detail) and the result is reported in the second bar above the heatmap (GGI risk, color code: grey = high risk, black = low risk). The first bar above the cluster shows the histological grade (grade, color code: yellow = grade 2, grey = grade 3, black = grade 1). Samples in the dendogram are indicated with the FF_ID or FFPE_ID plus the Pair_ID.
(TIF)

Figure S7 **Density distribution of genewise correlation between FF and FFPE paired samples versus average expression of all probes in FFPE (A) samples and in FF samples (B).**
(TIF)

Figure S8 **Distribution of genewise correlation between FFPE and FF paired samples.** (**A**) Density against the Pearson correlation distribution of all probes in FFPE and FF paired samples. (**B)** Frequency against the Pearson correlation distribution of all probes in FFPE and FF paired samples.
(TIF)

Figure S9 **Expression distribution of the top and bottom 500 correlating probes in FFPE samples (A) and in FF samples (B).** Density distribution of top 500, bottom 500 and all probes are displayed. Similar plots were obtained using the top 1000 and bottom 1000 probes (data not shown).
(TIF)
