# Supplementary figures and images for: Gene Expression Profiles from Formalin Fixed Paraffin Embedded Breast Cancer Tissue Are Largely Comparable to Fresh Frozen Matched Tissue
Source: PLoS One. 2011 Feb 11;6(2):e17163. doi: 10.1371/journal.pone.0017163 (PMC3037966; doi:10.1371/journal.pone.0017163)

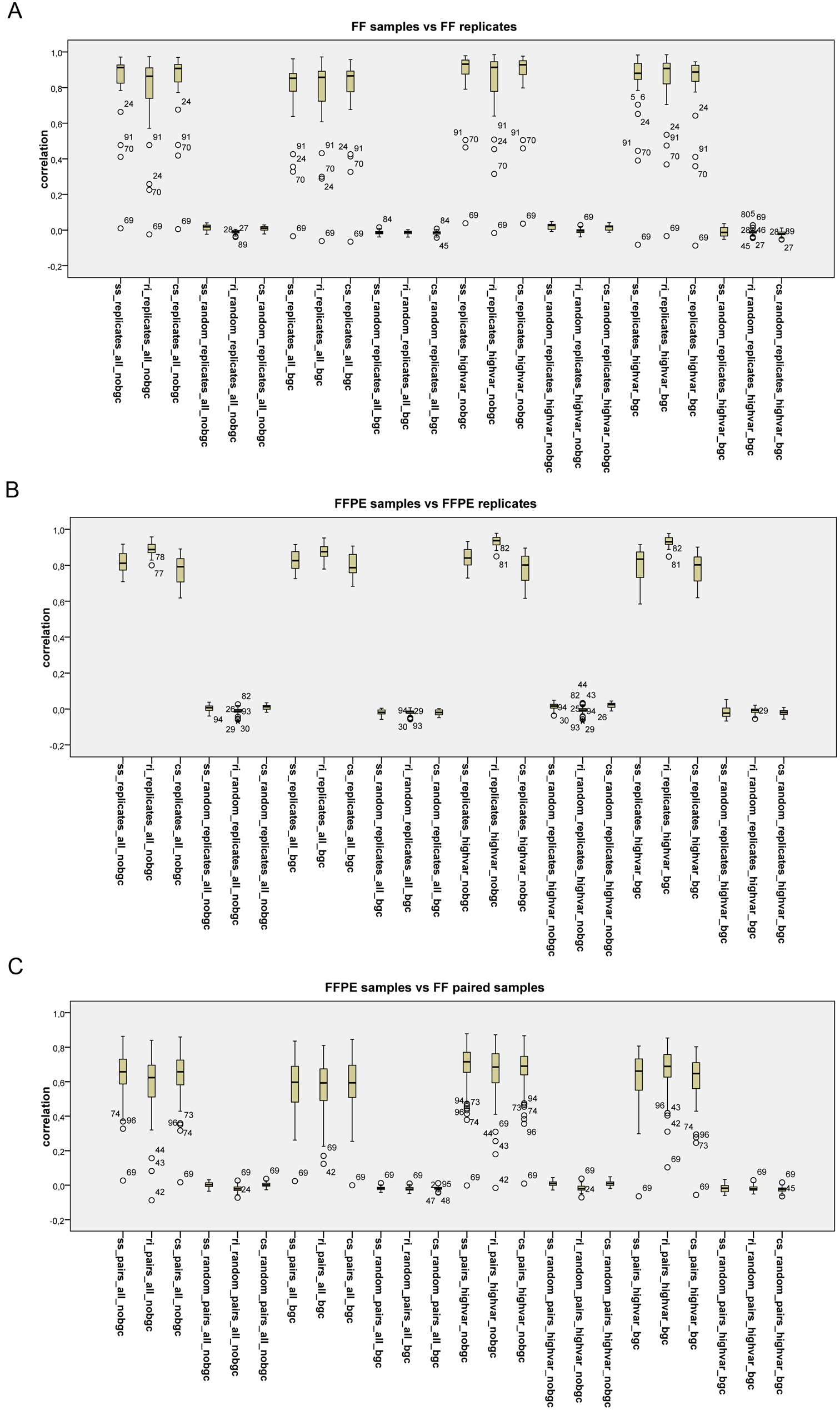

Supplement: Figure S1 — Box plots of the Pearson correlation coefficient between FF replicates (A), FFPE replicates (B) and FFPE/FF pairs (C) using three normalization procedures: simple scaling (ss), rank-invariant (ri) and cubic spline (cs) and carried out for either with background correction (bgc) or without background correction (nobgc). The box plots were generated using all probes that passed the p-value filtering (all) or using the informative probes (highvar). The number of informative probes using ss normalization without bgc is equal to 5480, with bgc is equal to 5284. The number of informative probes using ri normalization without bgc is equal to 6351, with bgc is equal to 6323. The number of informative probes using cs normalization without bgc is equal to 5388, with bgc is equal to 5664. In Panels A, B we report the correlation distribution of the comparison between real replicates (replicates) and between non-replicate samples (random_replicates). In Panel C we report the correlation distribution of the comparison between real pairs (pairs) and between non-paired samples (random_pairs). The circles in the graph are outlier samples indicated with the array_ID (see Table 1). The legend on the horizontal axis is a concatenation of the abbreviations for the normalization, sample pairing, probe set employed and the type of background correction applied. (TIF) [file pone.0017163.s001.tif]

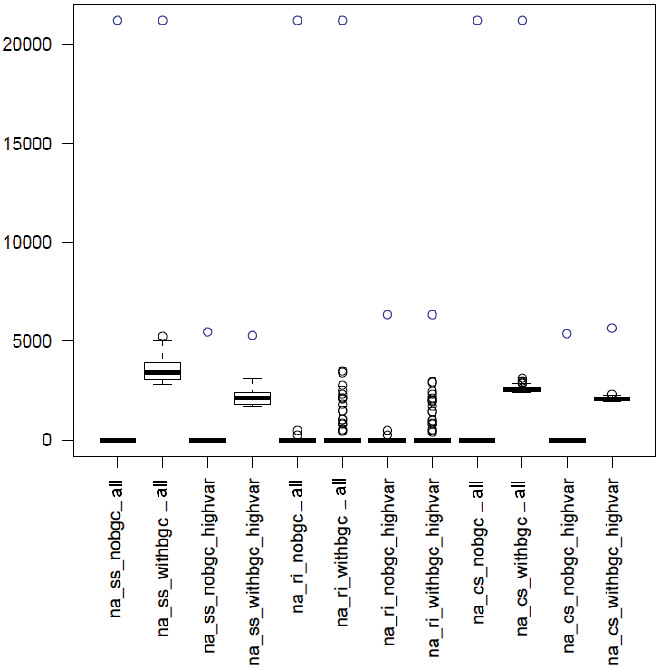

Supplement: Figure S2 — Number of probes on the array with negative values (na) after application of different normalization methods. The x-axis displays the normalization method (ss = simple scaling, ri = rank invariant, cs = cubic spline, nobgc = without background subtraction, withbgc = with background subtraction) and the dataset used (all = all probes that passed the p-value filtering, highvar = only informative probes). The y-axis represents the number of probes. The box plots represent the distribution of the number of probes with negative values for each option evaluated. If present, outlier samples are indicated with a black circle. The total number of probes in the dataset is represented by the blue circles. (TIF) [file pone.0017163.s002.tif]

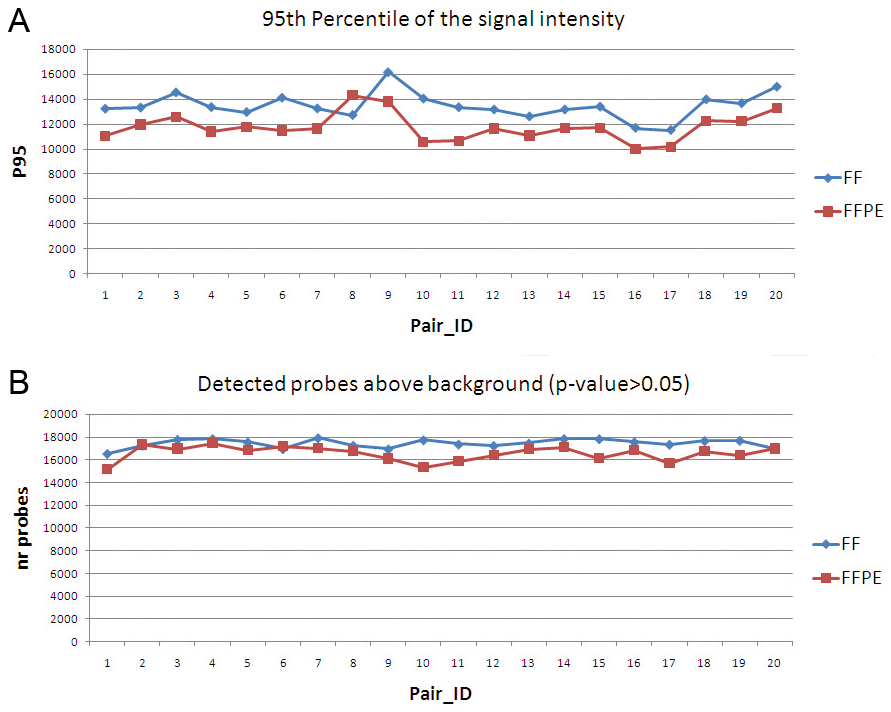

Supplement: Figure S3 — Quality control of the DASL gene expression data. (A) 95th percentile of the fluorescence intensity in FFPE and FF paired samples. On the x-axis we report the Pair_ID (1-20) and on the y-axis the 95th percentile (P95) of the fluorescence intensity. (B) Number of detected probes above background in FFPE and FF paired samples. On the x-axis we report the Pair_ID (1-20) and on the y-axis the number of probes significantly detected above background (p-value>0.05). (TIF) [file pone.0017163.s003.tif]

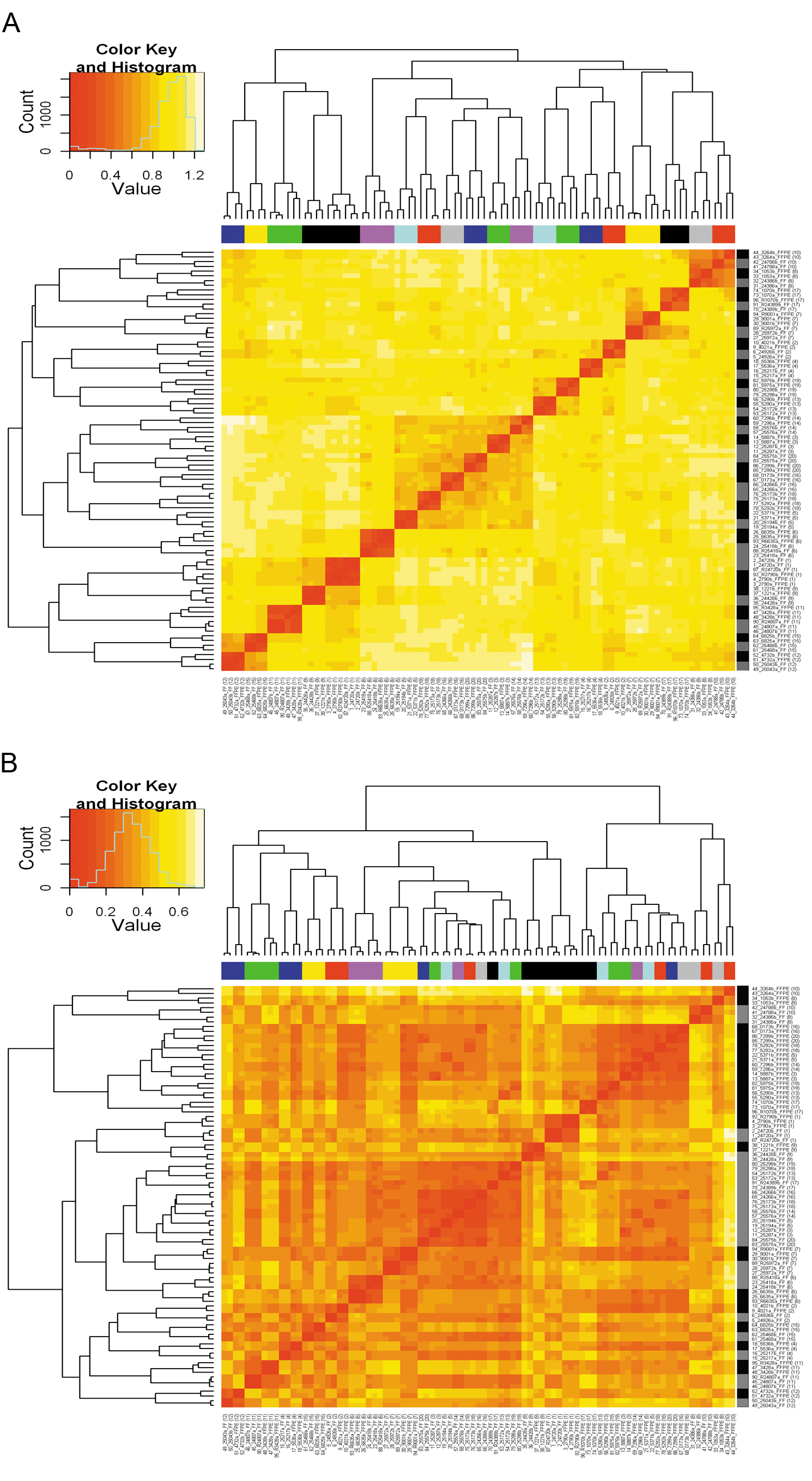

Supplement: Figure S4 — Heat maps of the distance measures (1-Pearson correlation coefficient) of the FFPE and FF samples (n = 89) using the informative probes (n = 5444). (A) Heat map using median centered log2 normalized data. Displayed are the distance measures of the FFPE and FF samples using all probes for calculating the distance sample by sample. Data are normalized with a simple scaling normalization without background correction and then median centered per probe, separately for FFPE and FF samples. Distances range from 0 (minimum distance) to 1.2 (maximum distance) as shown in the top left panel. The paired samples are indicated with a number from 1 to 20 and are color coded. (B) Heat map using non-median centered log2 normalized data. Displayed are the distance measures of the FFPE and FF samples using 5444 for calculating the distance sample by sample. Data are normalized with a simple scaling normalization without background correction. Distances range from 0 (minimum distance) to 0.6 (maximum distance) as shown in the top left panel. (TIF) [file pone.0017163.s004.tif]

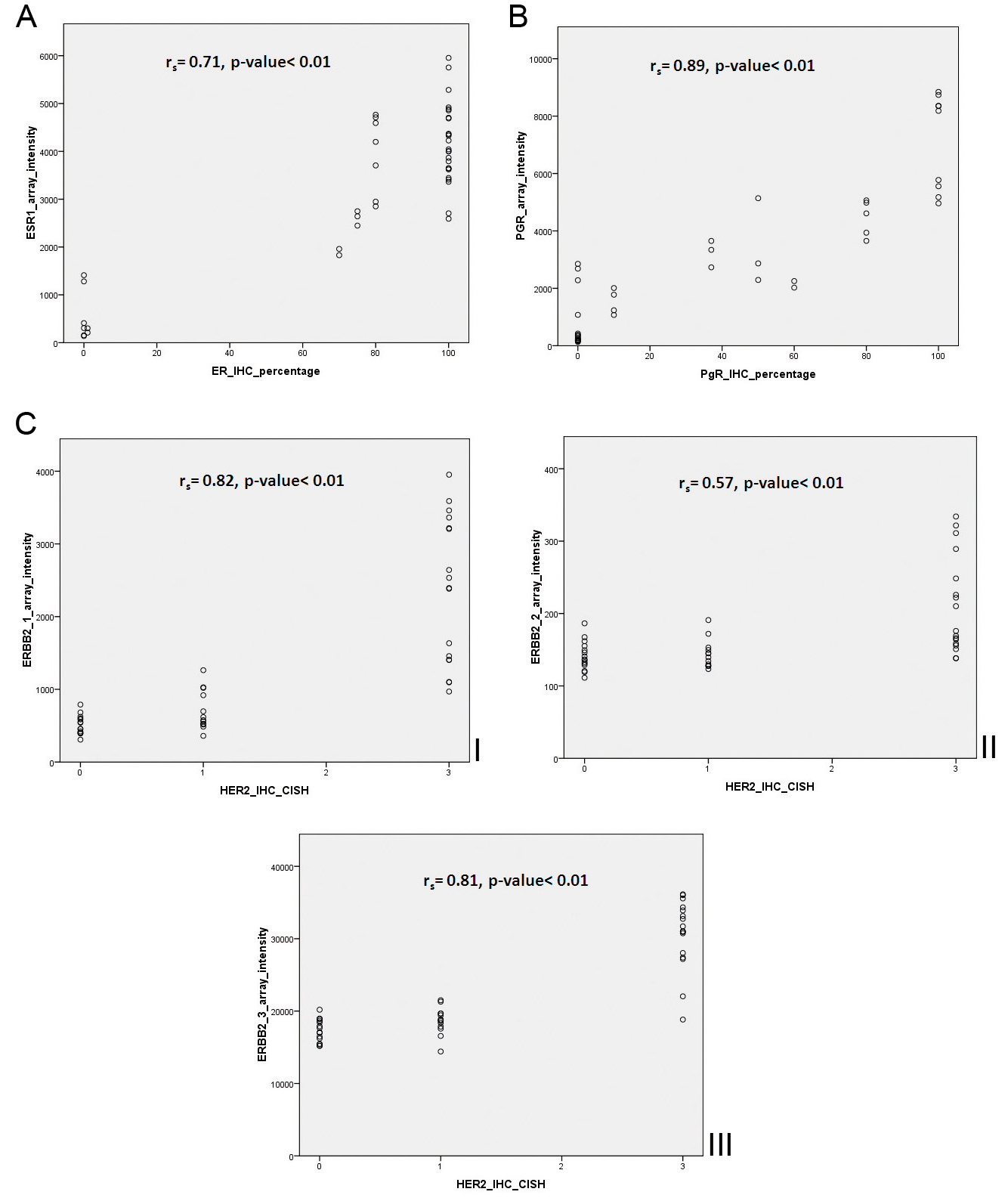

Supplement: Figure S5 — Comparison between the Immunohistochemistry (IHC) marker status (ER, PgR, HER2) and the gene intensity on the array (ESR1, PGR, ERBB2) for the FFPE samples (n = 45). (A). Dot plot of ER/ESR1: The x-axis reports the IHC percentage of staining of ER; the y-axis reports the absolute intensity of the gene ESR1 on the array. (B) Dot plot of PgR/PGR: The x-axis reports the IHC percentage of staining of PgR; the y-axis reports the absolute intensity of the gene PGR on the array. (C) Dot plots of HER2/ERBB2: The x-axis reports the IHC-CISH (Chromogenic In Situ Hybridization) status of HER2; the y-axis reports the absolute intensity of the gene ERBB2 represented by three different probes on the array (I, II, III). (TIF) [file pone.0017163.s005.tif]

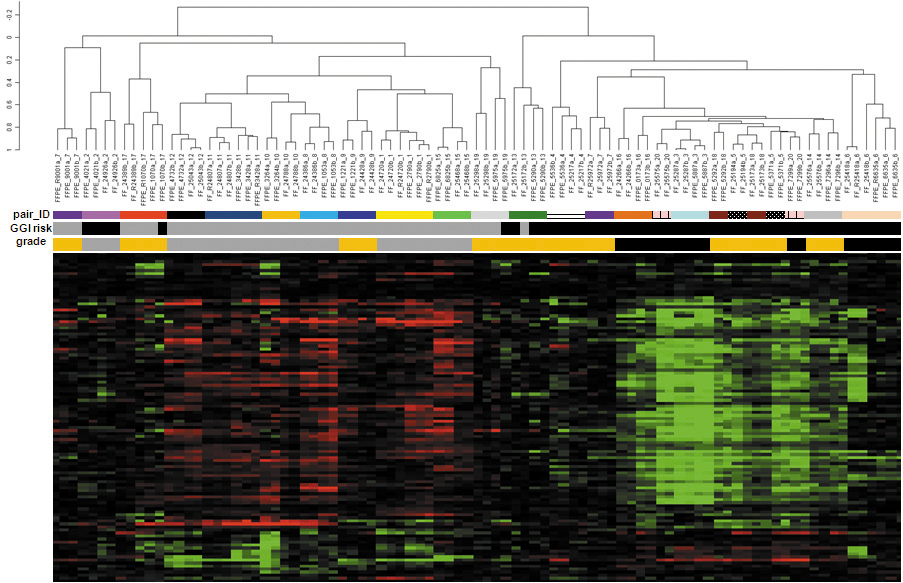

Supplement: Figure S6 — Unsupervised hierarchical clustering of all FFPE and FF samples (n = 89) with 110 DASL probes matching with the Genomic Grade Index (GGI) gene list. The clustering is performed using Pearson correlation and average linkage. The paired samples are color coded (pair_ID). Samples were classified as low risk or high risk based on the GGI score (see Methods for detail) and the result is reported in the second bar above the heatmap (GGI risk, color code: grey = high risk, black = low risk). The first bar above the cluster shows the histological grade (grade, color code: yellow = grade 2, grey = grade 3, black = grade 1). Samples in the dendogram are indicated with the FF_ID or FFPE_ID plus the Pair_ID. (TIF) [file pone.0017163.s006.tif]

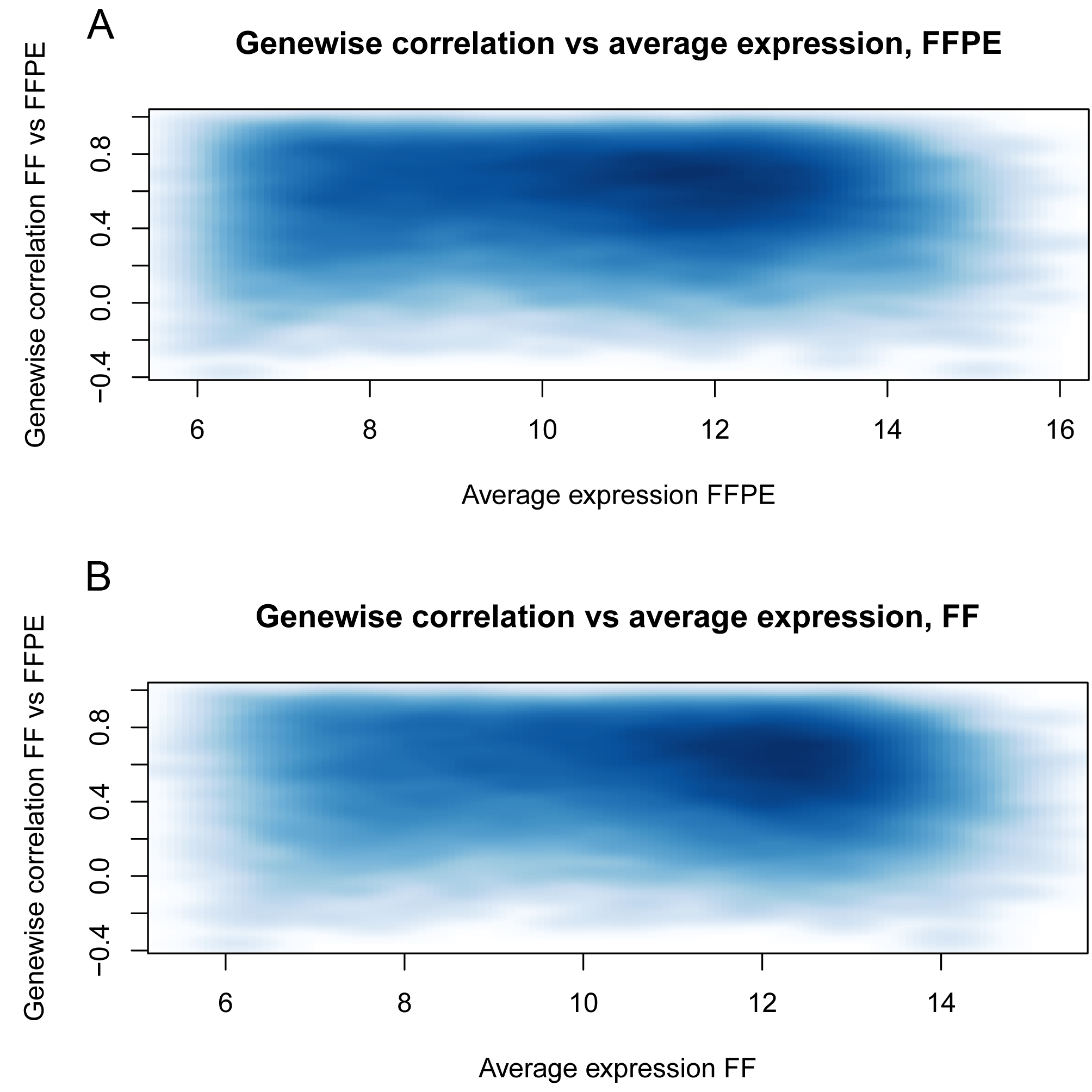

Supplement: Figure S7 — Density distribution of genewise correlation between FF and FFPE paired samples versus average expression of all probes in FFPE (A) samples and in FF samples (B). (TIF) [file pone.0017163.s007.tif]

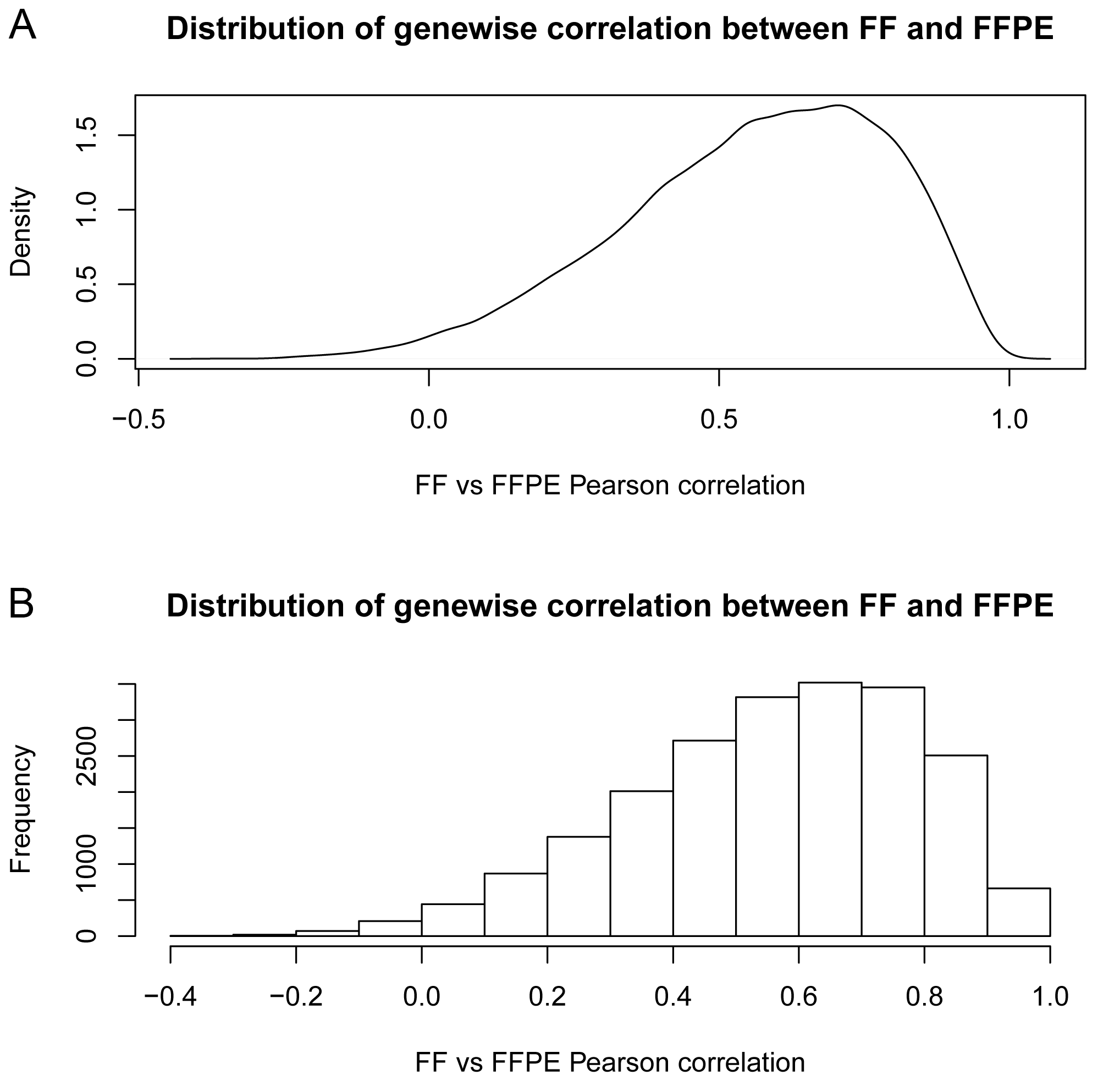

Supplement: Figure S8 — Distribution of genewise correlation between FFPE and FF paired samples. (A) Density against the Pearson correlation distribution of all probes in FFPE and FF paired samples. (B) Frequency against the Pearson correlation distribution of all probes in FFPE and FF paired samples. (TIF) [file pone.0017163.s008.tif]

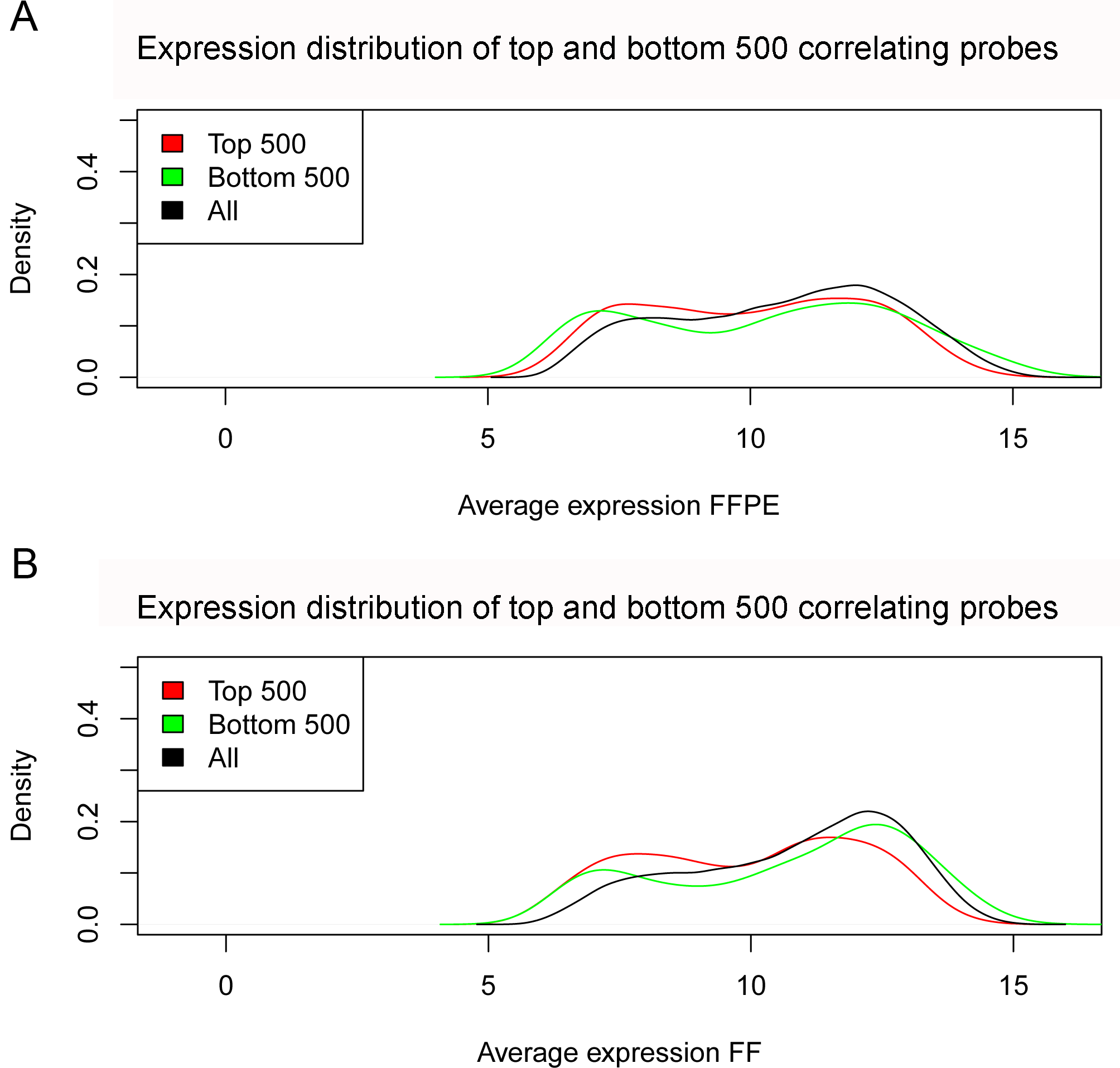

Supplement: Figure S9 — Expression distribution of the top and bottom 500 correlating probes in FFPE samples (A) and in FF samples (B). Density distribution of top 500, bottom 500 and all probes are displayed. Similar plots were obtained using the top 1000 and bottom 1000 probes (data not shown). (TIF) [file pone.0017163.s009.tif]
